# A Pilot Study Comparing the Effects of Consuming 100% Orange Juice or Sucrose-Sweetened Beverage on Risk Factors for Cardiometabolic Disease in Women

**DOI:** 10.3390/nu13030760

**Published:** 2021-02-26

**Authors:** Candice Allister Price, Valentina Medici, Marinelle V. Nunez, Vivien Lee, Desiree M. Sigala, Yanet Benyam, Nancy L. Keim, Ashley E. Mason, Shin-Yu Chen, Mariana Parenti, Carolyn Slupsky, Elissa S. Epel, Peter J. Havel, Kimber L. Stanhope

**Affiliations:** 1 Department of Molecular Biosciences, School of Veterinary Medicine, University of California Davis, Davis, CA 95616, USA; vilee@ucdavis.edu (V.L.); dmsigala@ucdavis.edu (D.M.S.); ybenyam@ucdavis.edu (Y.B.); pjhavel@ucdavis.edu (P.J.H.); klstanhope@ucdavis.edu (K.L.S.); 2 Division of Gastroenterology and Hepatology, Department of Internal Medicine, School of Medicine (V.M.), University of California Davis, Sacramento, CA 95817, USA; vmedici@ucdavis.edu; 3 Department of Nutrition, University of California Davis, Davis, CA 95616, USA; mvnunez@ucdavis.edu (M.V.N.); nancy.keim@ars.usda.gov (N.L.K.); sychen@mail.npust.edu.tw (S.-Y.C.); mgleal@ucdavis.edu (M.P.); cslupsky@ucdavis.edu (C.S.); 4 Western Human Nutrition Research Center, Agricultural Research Service, USDA, Davis, CA 95616, USA; 5 Osher Center for Integrative Medicine, School of Medicine, University of California San Francisco, San Francisco, CA 94155, USA; Ashely.Mason@ucsf.edu; 6 Department of Psychiatry, University of California San Francisco, San Francisco, CA 94143, USA; Elissa.Epel@ucsf.edu; 7 Basic Sciences, Touro University of California, Vallejo, CA 94592, USA

**Keywords:** fruit juice, sugar-sweetened beverage, uric acid, lipids, insulin sensitivity

## Abstract

Overconsumption of sugar-sweetened beverages increases risk factors associated with cardiometabolic disease, in part due to hepatic fructose overload. However, it is not clear whether consumption of beverages containing fructose as naturally occurring sugar produces equivalent metabolic dysregulation as beverages containing added sugars. We compared the effects of consuming naturally-sweetened orange juice (OJ) or sucrose-sweetened beverages (sucrose-SB) for two weeks on risk factors for cardiometabolic disease. Healthy, overweight women (*n* = 20) were assigned to consume either 3 servings of 100% orange juice or sucrose-SB/day. We conducted 16-hour serial blood collections and 3-h oral glucose tolerance tests during a 30-h inpatient visit at baseline and after the 2-week diet intervention. The 16-h area under the curve (AUC) for uric acid increased in subjects consuming sucrose-SB compared with subjects consuming OJ. Unlike sucrose-SB, OJ did not significantly increase fasting or postprandial lipoproteins. Consumption of both beverages resulted in reductions in the Matsuda insulin sensitivity index (OJ: −0.40 ± 0.18, *p* = 0.04 within group; sucrose-SB: −1.0 ± 0.38, *p* = 0.006 within group; *p* = 0.53 between groups). Findings from this pilot study suggest that consumption of OJ at levels above the current dietary guidelines for sugar intake does not increase plasma uric acid concentrations compared with sucrose-SB, but appears to lead to comparable decreases of insulin sensitivity.

## 1. Introduction

Consumption of sugar-sweetened beverages (SSB) at high levels is a well-established risk factor for obesity and cardiometabolic disease [1,2]. The added sugars most often used to sweeten SSBs are high-fructose corn syrup (HFCS)-55, which is 55% fructose and 45% glucose, or sucrose, which is 50% fructose and 50% glucose. Excess consumption of SSB leads to fructose overload in the liver, promoting numerous consequences [3], including upregulated *de novo* lipogenesis (DNL) [4] and uric acid production [5]. An increase in hepatic triglyceride accumulation leads to increased production and secretion of very low density lipoprotein (VLDL) [6], resulting in dyslipidemia. Hyperuricemia, best known for its contribution to gout [7], also plays a major role in the development of hypertension, and possibly atherosclerosis, chronic kidney disease, and type 2 diabetes (T2D) [8,9,10].

A commonly asked question is whether juice is better for health than SSB. Naturally-sweetened beverages (i.e., 100% fruit juice) do not contain added sugars, but still contain glucose and fructose as well as sucrose, with a fructose content approximately 80% to 130% of that found in SSBs [11]. Similar fructose content in naturally-sweetened fruit juice and SSBs, such as sodas, allows for the assumption that fruit juice is just as unhealthy to consume as SSBs. However, unlike SSBs, fruit juice contains many potentially beneficial bioactives that are thought to have protective health effects. These include bioavailable compounds such as polyphenols and vitamins [12,13]. Epidemiological studies have yielded conflicting results regarding the health effects of consuming 100% fruit juice. Some have shown that consumers of 100% fruit juice have lower body mass index (BMI), higher insulin sensitivity and lower odds of metabolic syndrome compared to non-consumers of 100% juice consumers [14,15,16,17]. Others have associated increased fruit juice consumption with increased incidence and risk of T2D [18,19] and metabolic syndrome [20]. It is important to note that “fruit juice” in the report by Muraki and colleagues [19] included both sugar-sweetened and naturally-sweetened juice, thus its conclusions are not specific to 100% fruit juice.

Orange juice (OJ) is the most commonly consumed fruit juice in the United States, but its effects on cardiometabolic health outcomes have not been rigorously studied [21]. Clinical intervention studies comparing naturally-sweetened beverages with SSBs on risk factors for cardiometabolic disease are scarce. To our knowledge, only four trials exist, two of which focused on OJ [22,23,24,25]. The longest of these studies [25] was a 12-week, randomized, placebo-controlled intervention in subjects who were overweight or obese. Subjects consuming OJ exhibited no detrimental effects on metabolic outcomes (e.g., insulin sensitivity, lipids, lipoproteins), however neither did the subjects consuming the energy-matched, sugar-sweetened control beverage. This is likely due to the very low levels of OJ and SSB consumption, representing only ~4% of daily energy requirements. Similar findings were observed in a 4-week intervention in which OJ was provided at ~7% of daily energy requirements, with the exception that improvements of blood pressure and fasting uric acid concentrations with OJ consumption compared with SSB consumption were observed [24].

The World Health Organization recommends reducing the intake of free sugars to less than 10% of total energy intake, and includes sugars naturally present in fruit juice as free sugars [26]. This is in contrast to the 2015–2020 Edition of the Dietary Guidelines for Americans, which recommends consuming less than 10% of calories per day from added sugar and does not include sugars naturally present in fruit juice as added sugars [27]. These contradictory guidelines reflect a lack of consensus regarding the effects of 100% fruit juice on metabolic health, undoubtedly due to the conflicting epidemiological evidence and the limited evidence from dietary intervention studies. We conducted a pilot study to compare the effects of consuming naturally-sweetened OJ or sucrose-sweetened beverages (sucrose-SB) on a number of risk factors for cardiometabolic disease.

## 2. Materials and Methods

### 2.1. Study Design

This was a parallel-arm, diet intervention study with 3 phases: (1) a two-day, 30-h inpatient baseline period during which participants resided at the inpatient facility located at the United States Department of Agriculture (USDA) Western Human Nutrition Research Center (WHNRC) on the University of California, Davis campus, consumed a standardized diet, and participated in experimental procedures (serial blood collections and an oral glucose tolerance test); (2) a 12-day outpatient intervention period during which participants consumed 3 servings/day OJ or sucrose-SB that provided 25% of daily energy requirement (Ereq) (calculated by the Mifflin equation [28]) as carbohydrate along with their usual *ad libitum* diets; (3) a 30-h inpatient intervention period during which participants consumed a standardized diet that included the beverages and repeated participation in the experimental procedures. This study was approved by the University of California, Davis Institutional Review Board and all subjects provided informed consent to participate in the study.

### 2.2. Study Participants

Women who were overweight or obese (BMI of 26 to 35 kg/m^2^), ages 25 to 40 years, were recruited via online advertisements on the University of California, Davis Department of Nutrition website, craigslist and hard copy postings in the cities of Davis, Woodland and Sacramento. We restricted this pilot study to a single sex because we and others have previously reported sex differences in the metabolic responses to sugar-sweetened beverages [5,29,30] and we did not have the resources to adequately power this study to test for a potential effect of sex. Exclusion criteria included evidence of diabetes or pre-diabetes (fasting glucose >100 mg/dL based on American Diabetes Association criteria [31]); triglycerides (TG) >200 mg/dL and low-density lipoprotein cholesterol (LDL-C) >130 mg/dL in combination with cholesterol:high-density lipoprotein (HDL-C) ratio >4 [32]; evidence of any other metabolic disorders (thyroid, kidney, etc.), pregnant or lactating, and use of tobacco. Participants were screened for eligibility by a 30-min phone interview, followed by a one-hour in-person screening interview at which time potential participants signed the consent form. Participants were instructed to fast for 12 h prior to their in-person interview for the collection of a fasting blood sample. Blood samples were measured for clinical chemistry, complete blood count (CBC), and lipids to determine eligibility. For the 5 weeks before the start of the study, participants were asked to limit daily consumption of sugar-containing beverages to no more than one 8 oz. (236 mL) serving of 100% fruit juice and to discontinue consumption of any vitamin, mineral, dietary, or herbal supplements. Participants were assigned to one of the two beverage groups matched for BMI. They were also matched for fasting plasma concentrations of TG, cholesterol, LDL-C and HDL-C assessed in the blood sample collected during screening. A total of 23 women were enrolled and 3 dropped or were dismissed from the study: one participant revealed withholding disqualifying information to study staff, another did not comply with study meal pick-ups and the third dropped due to a family emergency. A total of 20 participants (10/group) completed the study. Due to budgetary constraints, the sample size was set as the minimum number of women required to show significant effects of consuming 25% energy requirement (Ereq) as sucrose-SB for 2 weeks on risk factors. Ten subjects can detect an effect size of 0.9 in a paired comparison. Our preliminary data from a previously conducted study of 12 women consuming 25% Ereq from sucrose-SB for 2 weeks showed effect sizes ranging from 0.9–1.5 for the following risk factors: postprandial TG and apolipoprotein B (ApoB), fasting and postprandial non-high-density lipoprotein cholesterol (non-HDL-C), LDL-C, apolipoprotein CIII [ApoCIII], and uric acid.

### 2.3. Study Beverages

During intervention, subjects consumed 25% of Ereq as the available carbohydrate in OJ or sucrose-SB each day for 2 weeks. A dose of 25% of Ereq as sucrose-SB was selected as the positive control because unpublished results from our previous study showed that subjects consuming 25% Ereq sucrose-SB for 2 weeks exhibited significant increases in risk factors. It was our intent to ensure that the positive control beverage would increase risk factors. Previous studies utilizing lower doses of sucrose-SB as a control beverage failed to detect differences between OJ and sucrose-SB on risk factors [24,25], but also failed to detect within-group effects of sucrose-SB on risk factors [25]. Thus, the results did not provide insights as to whether consumption of naturally-occurring sugar in OJ leads to the same detrimental health effects as consumption of added sugar in sucrose-SB.

Each subject’s Ereq was calculated by the Mifflin equation with a physical activity adjustment of 1.5 for outpatient days and 1.3 for the inpatient days. The orange juice provided (Tropicana Homestyle^®^, light pulp) was purchased from local grocery stores, and consists of 90% of energy as available carbohydrate, 8% as protein and 2% as fat. Per 100 g, OJ contained 10 g of carbohydrate as approximately 4.2 g sucrose, 2.4 g fructose, 2.2 g glucose, and 1.2 g oligosaccharide (University of Minnesota Nutrition Data System for Research). The sucrose-SB were prepared as 10% C&H^TM^ cane sugar in water (*w*/*w*) and flavored with KoolAid^®^ (Kraft Heinz, Chicago, Illinois, USA). Thus, 100 g of sucrose-SB contained 10 g of sucrose. Both beverages were aliquoted into disposable, single serving bottles. During the outpatient days, participants were provided with three daily servings of sucrose-SB or OJ/day to be consumed one with each meal. The sucrose-SB and OJ provided were 1492 ± 48g and 1397 ± 43g per day, respectively, during the outpatient period. Subjects were instructed to consume the study beverages and no other sweetened beverages, including fruit juice and sweetened coffees or teas. They were also instructed to maintain their usual diets, eating as much or as little as they wished. The consent forms stated that the beverages contained a biomarker and that urine would be collected and analyzed for the biomarker to confirm that the study beverages were being consumed. Urine was collected at the specified time-points. However, due to problems with its solubility in the orange juice, the biomarker (riboflavin) was not added to the beverages.

#### Standardized Isocaloric Meals

On the day of and prior to the baseline and intervention inpatient experimental procedures at the WHNRC, subjects consumed provided staff-prepared meals. These meals were formulated to provide each individual’s Ereq (Mifflin with physical activity adjustment of 1.5 for outpatient consumption, 1.3 for inpatient consumption). The baseline meals contained 55% Ereq mainly as low-fiber complex carbohydrate (e.g., white bread, white rice, regular pasta), 30% from fat, and 15% from protein. The intervention meals were matched as closely as possible to the baseline meals except for the substitution of 25% of Ereq as carbohydrate in the beverages for complex carbohydrate in the meals. During the inpatient period, the sucrose-SB and OJ beverages provided were 1293 ± 42 g and 1210 ± 37 g per day, respectively. To mimic a typical daily incremental increase in energy intake in humans [33], the timing and the energy distribution of both the inpatient meals and beverages were as follows: breakfast—9:00-h/25%, lunch—13:00-h/35%, dinner—18:00-h/40%.

### 2.4. Metabolic Testing Procedures and Sample Collection

**Figure 1:**
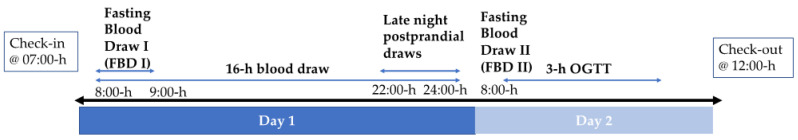
Schematic of 2-day inpatient visits. Oral glucose tolerance test (OGTT).

Body weight and blood pressure were measured during outpatient beverage and meal pick-up appointments and following 7:00-h check-in at the WHNRC inpatient facility. Percent body fat was measured by dual x-ray absorptiometry (DEXA) following check-in at baseline. At 7:30-h, after check-in, an IV catheter was inserted in an arm vein by a Registered Nurse and kept patent with slow (maximum = 20 mL/h) saline infusion. Figure 1 depicts the inpatient visit timeline, which is applicable to both baseline (week 0) and intervention (week 2) visits. A 24-h blood collection period began at 8:00-h (day 1 of 24-h blood collection). In total, three fasting blood samples were collected at 8:00-h, 8:30-h, and 9:00-h and the mean of these 3 samples represent fasting blood draw I (FBD I). Serial blood samples were collected at 30- or 60-min intervals from 9:30-h on day 1 to 8:00-h on day 2. Late-night postprandial concentrations reported here were collected at 22:00-h, 23:00-h, and 24:00-h on day 1. The day 2 8:00-h timepoint served as both the day 2 fasting blood draw (fasting blood draw II (FBD II)) and the 0-h timepoint for the oral glucose tolerance test (OGTT). This was followed by oral ingestion of 75 g glucose in 300 mL water and blood sampling 30, 60, 90, 120, and 180 min later. Catheter failures occurring in more than half of the participants resulted in missing data during the last 8-h of the 24-h blood collection period. Therefore, results from the first 16-h of blood collection are reported. In addition, two subjects (one OJ and one sucrose-SB) did not participate in the OGTT. All samples collected (e.g., plasma, urine, etc.) were stored at −80 °C.

Sample analyses: Plasma concentrations of TG, uric acid, glucose, and insulin were measured at all time points and the 16-h total area under the curves (AUC) were calculated by the trapezoidal method. The concentrations of cholesterol, LDL-C, non-HDL-C, and ApoB were measured during the fasting and late-night postprandial states. Fasting outcomes reported are from pooled plasma collected at 8:00-h, 8:30-h, and 9:00-h. Late-night postprandial outcomes reported are from pooled plasma collected at 22:00-h, 23:00-h, and 24:00-h [4] and will be referred to as “postprandial”. Lipid, lipoprotein, uric acid, and glucose concentrations were measured with a Polychem Chemistry Analyzer (PolyMedCo Inc., Anderson, SC, USA) with reagents from MedTest DX (Canton, MI, USA. Insulin was measured with radioimmunoassay (Millipore Inc., St. Charles, MO, USA). Insulin sensitivity was calculated by the Matsuda Index, an OGTT-derived index that uses both fasting and dynamic (postprandial) glucose and insulin concentrations [34]. Glucose and insulin total AUC during the OGTT were calculated by the trapezoidal method. Post-meal glucose and insulin amplitudes were calculated by subtracting the pre-meal glucose or insulin nadir from the post-meal peak concentrations. The mean amplitude was calculated for the 3 meals.

Plasma collected before (18:00-h) and after the dinner meal (19:00-h and 20:00-h) were used to measure pre- and post-meal metabolite concentrations. Due to budget constraints and sample availability, we were unable to conduct additional metabolite analyses utilizing samples collected after an overnight fast. Metabolite analysis was performed using nuclear magnetic resonance (NMR) spectroscopy, as previously described [35]. In brief, plasma was filtered through 3000 MW cutoff Amicon filters to remove protein and lipid particles. To 207 µL of filtrate, 23 µL of a 5 mM internal standard, 3-(trimethylsilyl)-1-propanesulfonic acid-d6, dissolved in 99.8% deuterium oxide, was added, and samples were transferred into 3 mm NMR tubes. NMR data were acquired on a Bruker AVANCE 600 MHz NMR spectrometer equipped with a SampleJet using the noesypr1d pulse sequence. Spectra were acquired at 25 °C, with a 2.5 s presaturation delay, a mixing time of 100 ms, an acquisition time of 2.5 s, 12 ppm sweep width, 8 dummy scans, and 32 transients. Spectra were processed and a total of 46 metabolites were identified and quantified using Chenomx NMRSuite v8.1 (Chenomx Inc., Edmonton, AB, Canada). Out of these 46 metabolites, we focused on 2-hydroxybutyrate, a biomarker of insulin resistance [36,37] and ethyl-β-d-glucuronide, a metabolite of ethanol [38].

### 2.5. Statistical Analyses

Anthropometric measures and metabolic characteristics at baseline were tested for differences between groups by a student *t*-test (SAS 9.3). Data were log-transformed when neither the baseline (week 0) nor absolute change (Δ) from baseline (week 2–week 0) values were normally distributed (Shapiro–Wilk: *p* < 0.05). The ∆outcome was analyzed in a primary analysis of covariance (ANCOVA) with adjustments for BMI and outcome at baseline. Covariates that did not improve the sensitivity of the model were removed. Within group changes from baseline were identified by least squares mean (LS mean) significantly different from zero. Secondary analyses were adjusted for baseline 16-h insulin AUC.

## 3. Results

### 3.1. Anthropometric Measures

Table 1 shows the participant characteristics at baseline, which were not significantly different between groups. Table 2 presents body weight, blood pressure, uric acid, lipid, lipoprotein and apolipoprotein measures at baseline (week 0) and at the end of the 2-week intervention (week 2) with the *p*-value for the effect of beverage group in the ANCOVA. There were no significant differences between the 2 groups in the changes of body weight or blood pressure at the end of the intervention. However, subjects consuming sucrose-SB exhibited a significant increase in body weight compared with baseline (sucrose-SB: +0.8 ± 0.4 kg, *p* = 0.03 within group; OJ: 0.6 ± 0.4 kg, *p* = 0.21 within group; *p* = 0.44 between groups).

### 3.2. Uric Acid

**Figure 2:**
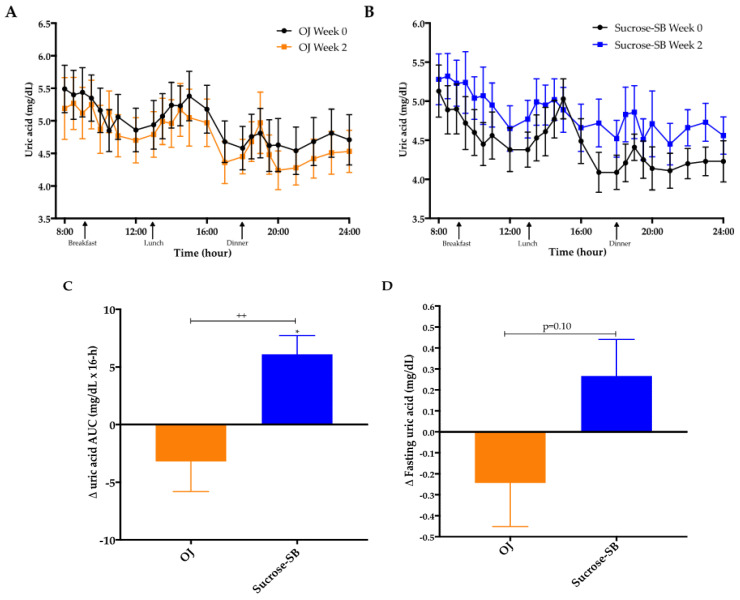
Uric acid concentrations: Sixteen-hour circulating uric acid concentrations before (black line) and after (orange line, orange juice (OJ); blue line, sucrose-sweetened beverage (sucrose-SB) consumption of either (**A**) OJ or (**B**) sucrose-SB for two weeks. Change of 16-h area under the curve (AUC) for (**C**) uric acid and (**D**) fasting uric acid in women consuming either naturally-sweetened OJ (orange bars) or sucrose-SB (blue bars) for two weeks. ++ *p* < 0.01, effect of group. * *p* < 0.05, LS mean of change different from zero.

Plasma uric acid concentrations over the 16-h blood collection period are presented for both baseline (week 0) and intervention (week 2) in Figure 2A,B. The 16-h uric acid AUC was increased in subjects consuming sucrose-SB compared with baseline (+6.2 ± 1.6 mg/dL × 16-h, *p =* 0.01 within group), and compared with subjects consuming OJ (−3.2 ± 2.6 mg/dL × 16-h, *p* = 0.15 within group; *p* = 0.008 between groups) (Figure 2C). The changes of fasting uric acid were not statistically different between groups (*p* = 0.10) (Figure 2D).

### 3.3. Plasma Cholesterol, Lipoproteins and Apolipoproteins

**Figure 3:**
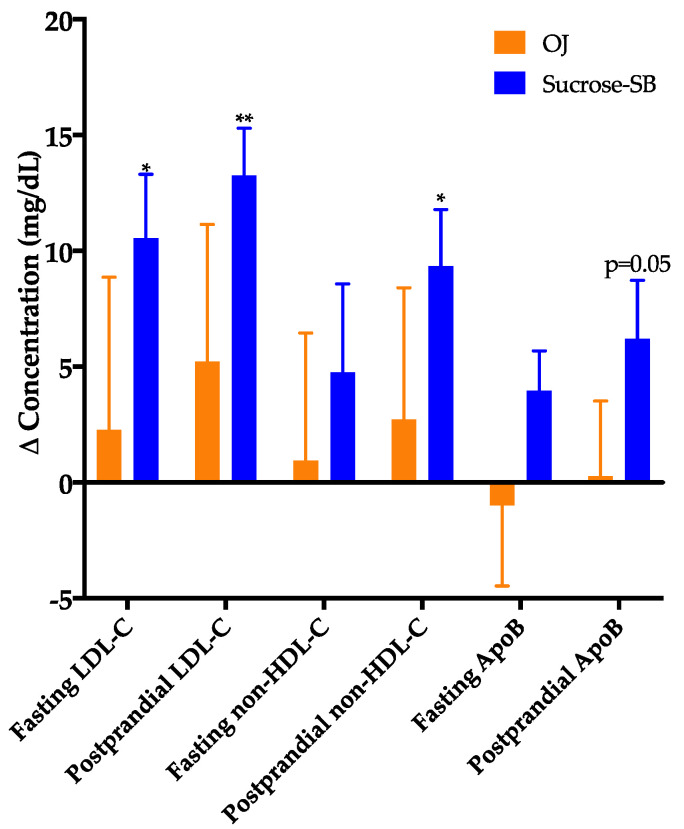
Changes of fasting and late-night postprandial low-density lipoprotein cholesterol (LDL-C), non-high-density lipoprotein cholesterol (non-HDL-C) and apolipoprotein B (ApoB) in women consuming either naturally-sweetened OJ (orange bars) or sucrose-SB (blue bars) for two weeks. * *p* < 0.05, ** *p* < 0.01, LS mean of change different from zero, primary ANCOVA.

The changes of fasting and late-night postprandial total cholesterol, LDL-C, non-HDL-C, or ApoB were not significantly different between the two groups (Figure 3), however, these outcomes indicated a pattern of being more adversely affected by sucrose-SB. Compared with baseline, fasting and postprandial LDL (*p* = 0.0498 and *p* = 0.006), postprandial ApoB, non-HDL-C and cholesterol (*p* = 0.05, *p* = 0.03, and *p* = 0.03) were increased in subjects consuming sucrose-SB. Insulin sensitivity at baseline was highly variable among the subjects, with the Matsuda Index ranging from 0.98 to 12.7 and the 16-h insulin AUC ranging from 197 to 1644 µU/mL × 16-h. To ensure this variability in baseline insulin sensitivity did not skew the results, secondary ANCOVAs were conducted that included adjustment for baseline insulin AUC (Matsuda not used for adjustment because outcomes were missing for 2 subjects). This adjustment for 16-h insulin AUC resulted in more adverse effects of sucrose-SB consumption on these lipoprotein risk factors. The within group effect of sucrose-SB consumption on postprandial ApoB was significant (*p* = 0.03, within group) and the significance for both fasting and postprandial LDL-C increased (*p* = 0.03 and *p* = 0.003, within group, respectively). There were no significant between or within group differences in fasting and postprandial HDL concentrations (Table 2).

### 3.4. Circulating Triglycerides (TG) and Apolipoprotein CIII (ApoCIII)

**Figure 4:**
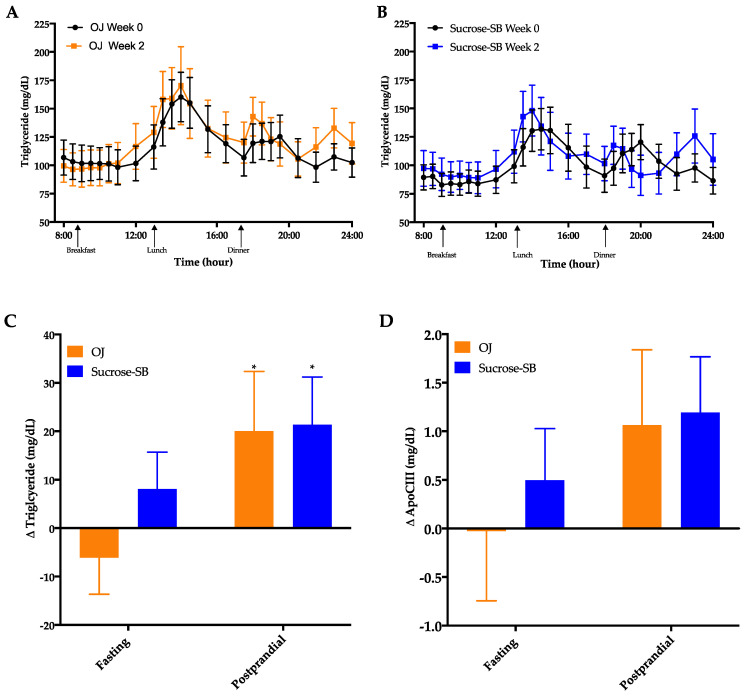
Sixteen-hour circulating triglyceride (TG) concentrations before (black line) and after (blue line) consumption of either (**A**) OJ or (**B**) sucrose-SB for two weeks. Changes in fasting and postprandial (**C**) triglyceride and (**D**) ApoCIII after two weeks of OJ or sucrose-SB consumption. * *p* < 0.05, LS mean of change different from zero. Apolipoprotein CIII (ApoCIII).

Circulating TG concentrations measured over the 16-h period are shown in Figure 4A,B. There were no significant within or between group differences in fasting TG (*p* = 0.24 between groups) or 16-h AUC (*p* = 0.89 between groups). However, late-night postprandial TG significantly increased in both groups (OJ: +20 ± 12 mg/dL, *p* = 0.019 within group; sucrose-SB: +21 ± 10 mg/dL, *p* = 0.038 within group; *p* = 0.82 between groups) (Figure 4C). Postprandial ApoCIII tended to parallel the postprandial TG data with both groups exhibiting increases after intervention, but these changes were not statistically significant (OJ: +1.1 ± 0.8, *p* = 0.14 within group; sucrose-SB: +1.2 ± 0.6, *p* = 0.10 within group; *p* = 0.88 between groups) (Figure 4D, Table 2).

### 3.5. Indices of Insulin Sensitivity and Post-Meal Glycemic Response

**Figure 5:**
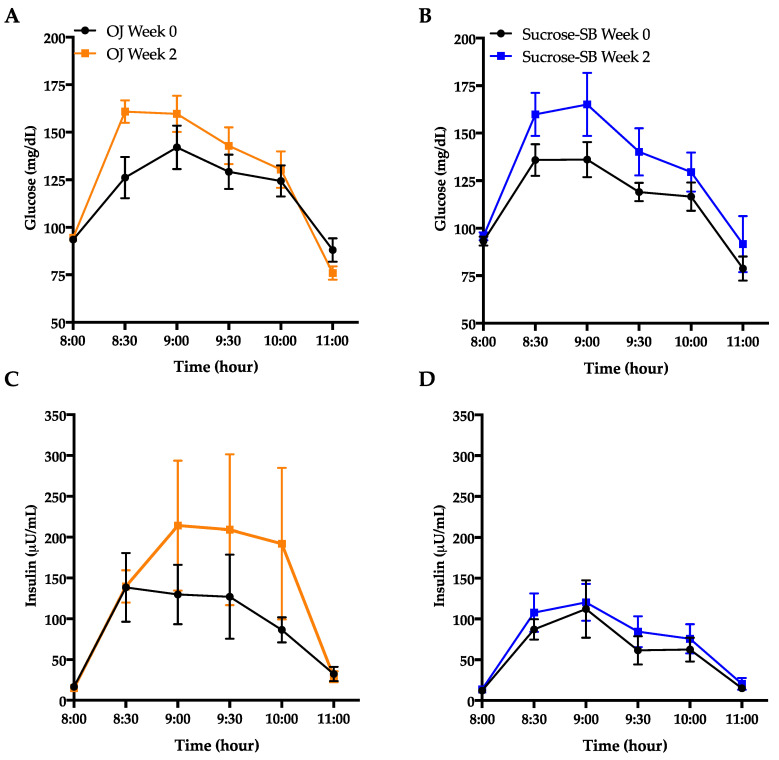

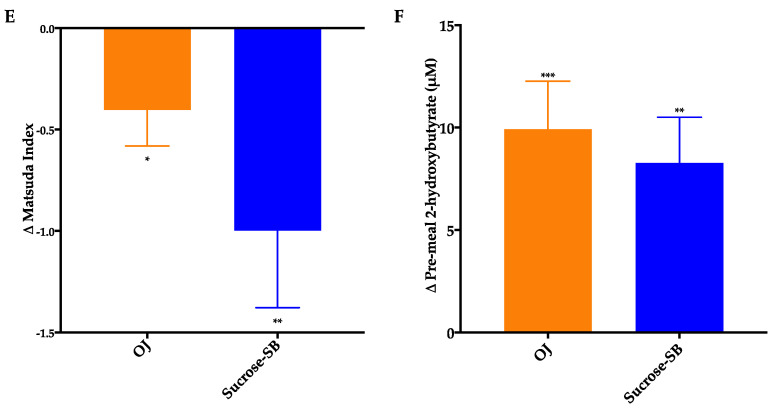
Metrics of insulin sensitivity measured by oral glucose tolerance test (OGTT) and the metabolite, 2-hydroxybutyrate. Glucose curves during the OGTT before (black line) and after (blue line) consumption of either (**A**) OJ or (**B**) sucrose-SB for two weeks; Insulin curves during the OGTT before (black line) and after (blue line) consumption of either (**C**) OJ or (**D**) sucrose-SB for two weeks; (**E**) Changes in Matsuda Index; (**F**) 2-hydroxybutyrate concentrations measured after a 4-h fast. * *p* < 0.05, ** *p* < 0.01, *** *p* < 0.001 LS mean of change different from zero.

Glucose and insulin excursions during oral glucose tolerance test (OGTT) are shown in Figure 5A–D. Indices of insulin sensitivity measured under fasting conditions and during an OGTT are reported in Table 3. Both groups exhibited decreases of insulin sensitivity calculated by the Matsuda Index (OJ: −0.40 ± 0.18, *p* = 0.04 within group; sucrose-SB: −1.0 ± 0.38, *p* = 0.006; *p* = 0.53 between groups) (Figure 5E). Glucose AUC during the OGTT significantly increased in the sucrose-SB group (+53.8 ± 22.5, *p* = 0.01 within group) but not in the OJ group (OJ: +31.7 ± 11.9, *p* = 0.10 within group; *p* = 0.44 between groups), whereas insulin AUC during the OGTT increased in the OJ group (+160.8 ± 92.2, *p* = 0.04) but not in the sucrose-SB group (+38.9 ± 36.8, *p* = 0.19; *p* = 0.53 between groups) (Table 3). Fasting glucose and insulin were measured at both the beginning (day 1, FBD I) and the end (day 2, FBD II) of the 24-h blood collection period. Compared with baseline, data from FBD I showed that fasting glucose increased in subjects consuming OJ (+4.6 ± 1.0 mg/dL, *p* = 0.02), but decreased in those consuming sucrose-SB (−3.8 ± 2.5 mg/dL, *p* = 0.05) (*p* = 0.006 between groups) (Table 3). However, 24-h later (FBD II) there were no changes compared with baseline in either group (OJ: +0.5 ± 1.0 mg/dL, *p* = 0.99; sucrose-SB: +0.70 ± 1.8 mg/dL, *p* = 0.61) and no difference between groups (*p* = 0.71). Similarly, fasting insulin measured from FBD I was significantly increased by OJ (+3.4 ± 1.6 µU/mL, *p* = 0.01), but did not change with sucrose-SB (+0.78 ± 0.80 µU/mL; *p* = 0.55) (*p* = 0.16 between groups) (Table 3). However, data from FBD II showed that neither group exhibited changes in fasting insulin (OJ: −0.20 ± 1.4 µU/mL, *p* = 0.75; sucrose-SB: +2.21 ± 0.2 µU/mL, *p* = 0.13 within group; *p* = 0.39 between groups). This variability was reflected in the homeostatic model assessment for insulin resistance (HOMA-IR), which significantly increased in participants consuming OJ using FBD I (OJ: +1.0 ± 044, *p* = 0.005; sucrose-SB; 0.08 ± 0.19, *p* = 0.91; *p* = 0.04 between groups), but was unchanged using FBD II (OJ: −0.007 ± 0.37, *p* = 0.90; sucrose-SB; +0.56 ± 0.17 µU/mL, *p* = 0.18; *p* = 0.41 between groups). Fasting concentrations of 2-hydroxybutryate, a biomarker of insulin resistance, significantly increased compared with baseline in both groups (OJ: +9.9 ± 2.3, *p* = 0.001; sucrose-SB: +8.3± 2.2, *p* = 0.006 within group; *p* = 0.66 between groups) (Table 3 and Figure 5F).

**Figure 6:**
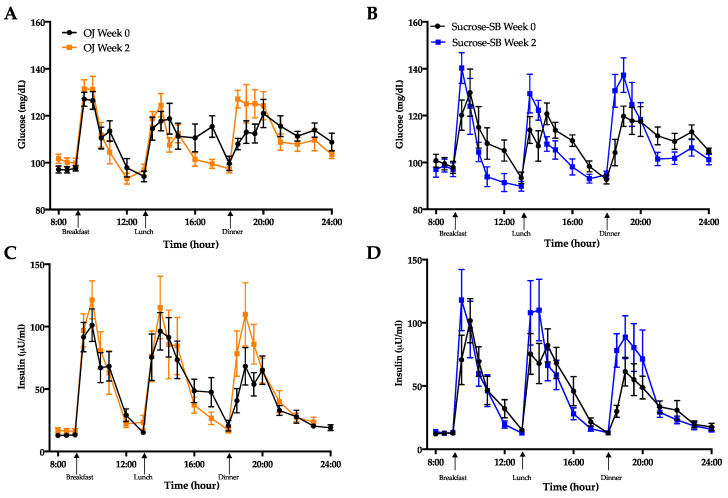
The 16-hour circulating glucose and insulin concentrations before (black line) and after (blue line) consumption of either (**A**,**C**) OJ or (**B**,**D**) sucrose-SB beverage for two weeks. Plasma was collected during consumption of energy-balanced meals at breakfast, lunch and dinner containing 55% Ereq as complex carbohydrate at baseline and during consumption of energy-balanced baseline diets containing 30% Ereq as complex carbohydrate and 25% as OJ or sucrose-SB at 2 weeks.

Circulating glucose and insulin concentrations measured over the 16-h period are presented in Figure 6A–D. As shown in Table 3, there were no within or between group differences for either the 16-h glucose or insulin AUC. The effects of the two beverages on post-meal glucose and insulin responses were also assessed (Table 3; Table 4). Post-meal glucose responses were increased by sucrose-SB consumption after all meals, thus the increase in mean post-meal glucose amplitude was higher in subjects consuming sucrose-SB than those consuming OJ. Post-breakfast, post-dinner, and mean post-meal insulin amplitudes were significantly increased by consumption of both beverages.

### 3.6. Ethyl-β-d-Glucuronide

**Figure 7:**
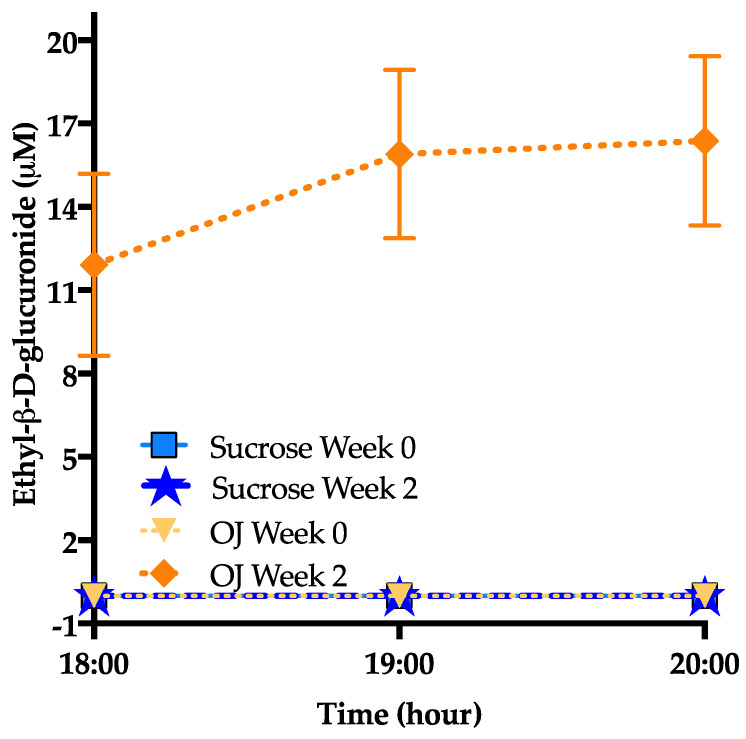
Baseline (week 0) and intervention (week 2) plasma ethyl-β-D-glucuronide before (18:00-h) and after (19:00-h and 20:00-h) consumption of an energy-balanced dinner. At baseline, dinner contained 55% Ereq as complex carbohydrate; at intervention, dinner contained 30% Ereq as complex carbohydrate and 25% as OJ or sucrose-SB.

As shown in Figure 7 ethyl-β-d-glucuronide, a metabolite of alcohol consumption [38] was elevated only in participants consuming OJ (+11.9 ± 3.3, *p* < 0.0001) but was undetected in those consuming sucrose-SB; (*p* = 0.001 between groups). This metabolite was undetected among38st all participants at baseline.

## 4. Discussion

Conflicting evidence concerning the health implications of consuming naturally-sweetened juice has led to contradictory dietary recommendations and guidelines. In this pilot study, we sought to address the question, “Does consumption of high levels of 100% fruit juice have similar effects on cardiometabolic risk factors as beverages containing added sugar (in this case sucrose)?” This pilot dietary intervention study is the first to compare consumption of naturally-sweetened orange juice with sucrose-SB at levels above dietary recommendations on cardiometabolic risk factors in overweight and obese women. Our findings indicate that, compared with sucrose-SB, consumption of OJ did not increase circulating uric acid. In addition, lipoproteins including LDL-C, non-HDL-C, and ApoB appeared to be less detrimentally affected by the consumption of OJ than sucrose-SB. However, consumption of both OJ and sucrose-SB modestly raised late-night postprandial TG concentrations and lowered insulin sensitivity.

Elevated uric acid is a well-known consequence of consuming fructose, sucrose, or HFCS-sweetened beverages [5,39,40,41] and our results add to this evidence base. Hepatic fructose overload results in AMP accumulation, which leads to increased uric acid production via the purine degradation pathway [42]. It has been previously reported that 4 weeks of OJ consumption lowered fasting uric acid concentration compared with sugar-SB [24]. Our results extend this finding, showing that compared with sucrose-SB, OJ protects against a sugar-induced increase in day-long uric acid concentrations even when consumed at levels above the dietary guidelines for free sugars. This may be in part due to the OJ having a slightly lower fructose content than the sucrose-SB (approximately 45 versus 50% fructose, respectively). In addition, it has been suggested that hesperidin, OJ’s main flavonoid (a type of polyphenol), may mediate the protective effects of OJ. In diabetic rats, hesperidin improves diabetic kidney dysfunction and inflammation [43] and reduces xanthine oxidase activity, the key enzyme in uric acid production [44,45,46]. Morand and colleagues tested hesperidin-specific effects by comparing OJ with sucrose-SB that was supplemented or not supplemented with hesperidin [24] in healthy men. Although they observed a protective effect of hesperidin on diastolic blood pressure, they found that the addition of hesperidin to sucrose-SB did not protect against increases in uric acid concentrations compared with sucrose-SB beverage without hesperidin. However, 100% OJ did prevent increases in uric acid compared to sucrose-SB. Thus, the authors attributed this protective effect of OJ to the presence of vitamin C. Previous studies have shown an inverse relationship between vitamin C and uric acid concentrations [47,48], including a meta-analysis reporting significant reductions in uric acid concentrations with vitamin C supplementation [49]. Studies to further investigate the inhibitory effects of the vitamin C and polyphenols contained in OJ on uric acid production are warranted.

Fasting and postprandial concentrations of LDL-C, non-HDL-C, and ApoB are established risk factors for cardiovascular disease [50]. Previous studies have shown that these risk factors are all increased in subjects consuming sucrose- or high-fructose corn syrup-SB [5,51,52,53], therefore the increases in lipoproteins that we observed in the women consuming sucrose-SB were expected. We did not observe a significant increase in these risk factors in the subjects consuming OJ. Previous studies showed that consuming OJ at 7% [54,55] or 10% [56] of calories resulted in a lowering of both total cholesterol and LDL in healthy participants. In another study, decreases of LDL were observed in 14 hypercholesteremic subjects consuming 10% of energy as OJ for 8 weeks, but not in 31 normocholesterolemic subjects [57]. These studies, along with our current results, suggest that OJ at low levels of consumption may have beneficial anti-atherogenic effects on lipoprotein and cholesterol risk factors, and at high levels of consumption, may have less detrimental effects than the same high levels of SSB. Data from in vitro experiments suggest the protective effects of OJ may be mediated by hesperidin and naringenin, which have been reported to inhibit cholesteryl ester and ApoB synthesis [58]. Interestingly, our results indicate that OJ and sucrose-SB induced significant and comparable increases of plasma TG concentrations during the postprandial sampling period. We have previously demonstrated that SSB consumption induces a second post-dinner peak, between 22:00 and 24:00-h, that is absent when complex carbohydrate is consumed [5]. The significant and comparable increase in postprandial TG concentrations exhibited by subjects consuming OJ is somewhat surprising given they did not also exhibit an increase in postprandial ApoB at these same timepoints. This suggests that consumption of OJ resulted in the secretion of fewer VLDL particles than sucrose-SB, but these fewer particles were more enriched with TG. It is also possible that OJ consumption resulted in secretion of fewer VLDL particles that were not TG-enriched, rather the TG in subjects consuming OJ was cleared more slowly. As this is the first study to report effects of OJ consumption on postprandial TG concentrations, more studies are needed to confirm these results and investigate the potential mechanisms.

In this pilot study, we also report that consumption of both 100% OJ and sucrose-SB decreased insulin sensitivity, as assessed by the Matsuda Index. To the best of our knowledge, this is the first study to assess insulin sensitivity in subjects consuming OJ utilizing an OGTT-derived index. Our results are in contrast to those of previous studies that reported improved insulin sensitivity in subjects consuming OJ when assessed by HOMA-IR [25,54,55]. It is possible that the improved [55] or unaffected [25] HOMA-IR observed in subjects consuming OJ in previous studies may relate to the amount of OJ consumed. The subjects in our study consumed more than twice the amount of OJ as the subjects in these studies [25,55]. However, we [59], and others [60] have previously suggested that HOMA-IR is a less reliable index of insulin sensitivity than the indices generated during hyperinsulinemia euglycemic clamps, OGTT or fast sample intravenous glucose tolerance tests. The inconsistent fasting glucose and insulin values observed during this study within a 24-h period supports this argument, with one set of values suggesting only OJ increased HOMA-IR and the other set suggesting neither beverage affected HOMA-IR. The equally significant increases of circulating concentrations of the hepatic metabolite, 2-hydroxybutyrate (also known as α-hydroxybutyrate) that occurred in both groups further support the reductions in insulin sensitivity observed in both beverage groups. Elevated concentrations of 2-hydroxybutyrate are reported to represent increased oxidative stress and predict impaired glucose tolerance [36]. Previous acute studies have demonstrated protective effects of flavonoids and vitamin C in OJ on oxidative stress and inflammation at doses above the dietary recommendation for sugar consumption but below the dose provided in our study [61,62]. Thus, our observation of increased 2-hydroyxbutyrate in those consuming OJ suggests that high and continuous consumption of OJ may override the protective effects of OJ on oxidative stress, possibly contributing to worsening glucose tolerance.

As indexed by the mean amplitude, subjects consuming sucrose-SB exhibited significantly higher mean glucose post-meal peaks compared with baseline and compared with subjects consuming OJ. This could be explained by the OJ polyphenol, naringenin, which has been shown to slow gastric emptying of glucose in animal models [63], but this has not yet been demonstrated in humans.

One interesting outcome of this study was the increase in ethyl-β-d-glucuronide, a well-known biomarker for monitoring ethanol intake [38,64], in the plasma of subjects consuming OJ. This metabolite was not observed at baseline and was not observed after intervention in participants consuming sucrose-SB. Ethanol is present in OJ at low levels (approximately 0.20 g up to 0.72 g per liter of OJ) [64], yet surprisingly the levels of ethyl-β-d-glucuronide detected after OJ consumption were comparable to that detected in people who report heavy drinking [65]. Notably, we observed a significantly higher level of plasma acetate in those consuming OJ after the 2-week intervention. Acetate has previously been shown to increase after alcohol consumption [66]. However, we did not observe differences in blood ethanol levels over those measured at baseline or between groups. Further work will be needed to determine the mechanisms explaining elevated ethyl-β-d-glucuronide after high OJ consumption.

The strengths of this study include the sucrose-SB control group, the provision of individualized portions of study beverage based on calculated Ereq, and the provision of standardized, eucaloric diets the day prior and the day of the 24-h blood collections. A further strength is the outcome assessments during both the fasting and postprandial period, as well as a 16-h period for several of the outcomes (uric acid, TG, glucose and insulin). Relative to the use of HOMA-IR, utilization of the Matsuda Index based on data from an OGTT is a strength of the study design. Limitations include only women who were overweight to obese were studied, thus results are not generalizable to male or lean subjects. Furthermore, we did not control for menstrual cycles, which could potentially affect outcomes such as TG concentrations. Other limitations include the small sample size and the short duration of the intervention, however this project was conceived and performed as a pilot study in order to obtain preliminary data in support of a larger, longer term study which is now underway. The ongoing study includes a larger sample size, both sexes, a longer intervention period, and the use of a hyperinsulinemic clamps to assess both hepatic and whole-body insulin sensitivity.

## 5. Conclusions

The results of this pilot study provide new and intriguing data showing that consuming 100% OJ provided at 25% of daily Ereq for two weeks did not increase plasma uric acid concentrations compared with the same amount of sucrose-SB and had little to no effect on plasma lipoproteins. However, both OJ and sucrose-SB increased postprandial TG and decreased insulin sensitivity. We also observed elevated plasma concentrations of ethyl-β-d-glucuronide in subjects consuming OJ but not in those consuming sucrose-SB. Additional studies are needed to determine the health effects of OJ and other 100% fruit juices compared to sugar-sweetened beverages.

## Figures and Tables

**Table 1:** Baseline Participant Characteristics.

	OJ	Sucrose	Effect of Group
	(*n* = 10)	(*n* = 10)	*P*
Age (years)	33.2 ± 1.2	31.2 ± 1.7	0.35
Body weight (kg)	80.1 ± 3.8	87.4 ± 3.8	0.44
Body mass index (kg/m^2^)	30.2 ± 1.0	31.0 ± 1.0	0.98
% Body fat	40.5 ± 2.1	40.2 ± 1.4	0.88
Waist circumference (cm)	88.3 ± 4.5	86.3 ± 2.4	0.70

Values are expressed as mean ± SEM. *p*-value < 0.05 indicates significant difference between groups by one-factor ANOVA. Orange juice (OJ).

**Table 2:** Body weight and cardiometabolic risk factors.

	OJ (*n* = 10)		Sucrose (*n* = 10)		Effect of Beverage
	Baseline Week 0	Intervention Week 2	Baseline Week 0	Intervention Week 2	
	Mean ± SEM	Mean ± SEM	Mean ± SEM	Mean ± SEM	*P*
Body weight (kg)	80.1 ± 3.8	80.8 ± 3.6	87.4 ± 3.8	88.2 ± 3.8 *	0.44
Systolic blood pressure (mmHg)	111.6 ± 2.1	115.3 ± 3.1	121.7 ± 4.2	121.8 ± 2.2	0.47
Diastolic blood pressure (mmHg)	71.5 ± 2.4	70.2 ± 2.7	69.7 ± 3.2	70.3 ± 2.4	0.88
Fasting uric acid (mgdL)	5.4 ± 0.4	5.2 ± 0.4	5.0 ± 0.3	5.3 ± 0.3	0.10
AUC uric acid (mg/dL × 16-h) ^2^	78.5 ± 5.6	75.3 ± 5.3	70.3 ± 3.8	76.5 ± 4.1 *	0.008
Fasting total cholesterol (mg/dL) ^2^	172.7 ± 8.7	174.7 ± 8.3	167.5 ± 12.3	174.9 ± 9.2	0.48
Postprandial total cholesterol (mg/dL) ^2^	163.5 ± 8.2	168.5 ± 7.1	158.8 ± 10.2	169.3 ± 9.4 *	0.40
Fasting LDL-C (mg/dL) ^2^	123.3 ± 9.9	126.8 ± 10.1	110.3 ± 8.3	120.8 ± 7.3 *	0.47
Postprandial LDL-C (mg/dL) ^2^	118.2 ± 9.5	124.3 ± 9.8	105.7 ± 6.6	118.9 ± 7.2 **	0.32
Fasting non-HDL-C (mg/dL)	133.7 ± 8.7	130.3 ± 7.9	119.4 ± 9.0	124.1 ± 6.9	0.62
Postprandial non-HDL-C (mg/dL) ^1^	122.2 ± 8.6	127.9 ± 6.9	112.2 ± 7.2	121.55 ± 7.4 *	0.71
Fasting ApoB (mg/dL) ^2^	63.2 ± 5.8	62.7 ± 5.9	54.0 ± 5.7	57.9 ± 4.9	0.35
Postprandial ApoB (mg/dL) ^2,3^	61.4 ± 6.2	62.6 ± 5.0	54.2 ± 5.4	60.4 ± 5.7 *	0.31
Fasting HDL (mg/dL)	43.4 ± 2.1	44.4 ± 2.4	48.2 ± 4.6	50.8 ± 4.4	0.42
Postprandial HDL (mg/dL)	41.3 ± 2.3	41.5 ± 2.6	46.6 ± 4.2	47.8 ± 3.8	0.43
Fasting TG (mg/dL)	103.9 ± 15.7	97.8 ± 15.0	87.6 ± 33.5	95.7 ± 14.8	0.24
Postprandial TG (mg/dL) ^1,2^	102.8 ± 12.3	122.8 ± 17.3 *	92.2 ± 12.7	113.6 ± 21.5 *	0.82
TG AUC (mg/dL × 16-h)	1855.4 ± 257.8	1971.0 ± 313.4	1606.11 ± 220.6	1713.0 ± 279.3	0.89
Fasting ApoCIII (mg/dL) ^2^	8.4 ± 1.0	8.4 ± 1.2	7.7 ± 1.2	8.1 ± 1.3	0.65
Postprandial ApoCIII (mg/dL) ^2^	7.6 ± 0.8	8.7 ± 1.2	7.5 ± 1.3	8.7 ± 1.5	0.97

Bolded *p*-value < 0.05 indicates significant difference between groups by one-factor analysis of covariance (ANCOVA) of 1transformed or untransformed data with adjustments for outcome at baseline and 2BMI and/or 3baseline 16-h insulin AUC. Values expressed as mean ± standard error of the mean (SEM). * *p*-value < 0.05, ** *p*-value < 0.01, LS mean of change different from zero. Orange juice (OJ), triglycerides (TG), low-density lipoprotein cholesterol (LDL-C), non-high-density lipoprotein cholesterol (non-HDL-C), apolipoprotein B (ApoB), high-density lipoprotein (HDL), apolipoprotein CIII (ApoCIII), area under the curve (AUC). Late-night postprandial blood collected at 22:00-h, 23:00-h and 24:00-h.

**Table 3:** Indices of insulin sensitivity.

	OJ (*n* = 10)		Sucrose (*n* = 10)		
	Baseline	Intervention	Baseline	Intervention	Effect of Beverage
	Week 0	Week 2	Week 0	Week 2	
	Mean ± SEM	Mean ± SEM	Mean ± SEM	Mean ± SEM	*P*
Matsuda Index ^1^	2.8 ± 0.4	2.5 ± 0.4 *	4.4 ± 1.1	3.4 ± 0.8 **	0.53
OGTT Glucose AUC (mg/dL × 180 min)	359.3 ± 16.6	391.0 ± 16.3	345.7 ± 11.7	399.5 ± 27.0 *	0.44
OGTT Insulin AUC (µU/mL × 180 min)	282.9 ± 78.8	443.6 ± 166.9 *	188.1 ± 40.5	226.9 ± 40.7	0.53
*Glucose, insulin and HOMA-IR from FBD I on day 1 of the 24-h blood collection period*					
Fasting glucose (mg/dL)	97.1 ± 1.5	101.7 ± 2.0 *	100.8 ± 2.8	97.0 ± 3.2	0.006
Fasting insulin (µU/mL)	13.1 ± 0.9	16.5 ± 2.2 *	12.5 ± 1.2	13.3 ± 1.5	0.16
HOMA-IR ^2^	3.1 ± 0.2	4.2 ± 0.6 **	3.1 ± 0.3	3.2 ± 0.4	0.04
*Glucose, insulin and HOMA-IR from FBD II on day 2 of the 24-h blood collection period*					
Fasting glucose (mg/dL)	98.7 ± 1.3	98.7 ± 1.5	97.1 ± 2.3	97.8 ± 2.6	0.70
Fasting insulin (µU/mL)	16.3 ± 1.3	16.1 ± 1.8	11.8 ± 1.5	14.0 ± 1.7	0.39
HOMA-IR ^2^	4.0 ± 0.3	4.0 ± 0.5	2.9 ± 0.4	3.4 ± 0.4	0.41
2-hydroxybutyrate (µM)	26.6 ± 2.3	37.1 ± 2.4 **	26.8 ± 3.2	36.4 ± 3.9 **	0.66
16-h Glucose AUC (mg/dL × 16-h)	1769.3 ± 35.0	1750.4 ± 35.0	1740.9 ± 39.4	1702.0 ± 39.4	0.38
16-h Insulin AUC (µU/mL × 16-h)	757.0 ± 10.91	820.8 ± 141.2	658.7 ± 99.3	711.7 ± 112.5	0.87
Mean Glucose AMP (mg/dL)	37.2 ± 1.4	41.7 ± 1.8	39.4 ± 3.4	55.0 ± 5.8 ***	0.04
Mean Insulin AMP (µU/mL)	85.1 ± 10.5	108.3 ± 18.4 **	79.4 ± 14.3	114.9 ± 20.6 ***	0.20

Amplitude (AMP) was calculated by subtracting the pre-meal nadir concentration from post-meal peak concentration for all subjects at each meal. Bolded *p*-value < 0.05 indicates significant difference between groups by one-factor ANCOVA of ^1^ transformed or untransformed data with adjustments for ^2^ BMI and/or outcome at baseline. * *p*-value < 0.05, ** *p*-value < 0.01, *** *p*-value < 0.001, LS mean of change different from zero. Oral glucose tolerance test (OGTT), area under the curve (AUC), homeostatic model assessment for insulin resistance (HOMA-IR).

**Table 4:** Changes in glucose and insulin amplitudes after two weeks of OJ or sucrose-SB intake.

	OJ	Sucrose	Effect of Beverage
	Mean ± SEM	Mean ± SEM	*P*
Glucose post-breakfast AMP (mg/dL)	3.0 ± 42	8.9± 6.1 *	0.14
Glucose post-lunch AMP (mg/dL)	0.75 ± 5.8	17.2 ± 8.9 *	0.20
Glucose post-dinner AMP (mg/dL)	9.6 ± 4.7	20.6 ± 6.7 **	0.16
Mean Glucose AMP (mg/dL)	4.5 ± 2.0	15.6 ± 4.4 ***	0.04
Insulin post-breakfast AMP (µU/mL)	20.8 ± 5.0 ***	26.7 ± 7.9 **	0.52
Insulin post-lunch AMP (µU/mL)	7.5 ± 12.4	31.8 ± 19.5	0.23
Insulin post-dinner AMP (µU/mL)	41.4 ± 18.0 *	48.1 ± 15.9 *	0.76
Mean Insulin AMP (µU/mL)	31.2 ± 12.8 **	42.5 ± 10.7 ***	0.50

Amplitude (AMP) was calculated by subtracting the pre-meal nadir concentration from post-meal peak concentration for all subjects at each meal. Bolded *p*-value < 0.05 indicates significant difference between groups by ANCOVA. * *p*-value < 0.05, ** *p*-value < 0.01, *** *p*-value < 0.001, LS mean of change different from zero.

## Data Availability

The data presented in this study are available on request from the corresponding author.
